# Enhancing Multidimensional Health Benefits Through the Use of Mobile Leisure Application

**DOI:** 10.3390/healthcare14020246

**Published:** 2026-01-19

**Authors:** Jae Hyung Park, Chul Won Lee, Chanwook Do

**Affiliations:** 1 Department of Sport Industry Studies, College of Educational Sciences, Yonsei University, Seoul 03722, Republic of Korea; pjayhsports@gmail.com (J.H.P.); wakeford@yonsei.ac.kr (C.W.L.); 2 Georgia Games, P.O. Box 2043, Kennesaw, GA 30156, USA

**Keywords:** smartphone-based leisure reservation platforms, Interpretative phenomenological analysis, physical health, mental health, social health

## Abstract

**Background/Objectives**: Smartphone-based leisure reservation platforms increasingly shape how individuals participate in leisure, yet little is known about how such technology-mediated engagement influences users’ awareness of multidimensional health benefits. The purpose of this study is to investigate how regular users of smartphone-based leisure reservation platforms perceive multidimensional health benefits associated with their leisure activities. **Methods**: Based on a constructivist/interpretivist approach, this study applied Interpretative Phenomenological Analysis (IPA). Ten participants with at least one year of platform use completed semi-structured interviews. Data were analyzed through iterative coding and theme development, with trustworthiness ensured through member checking, peer debriefing, and triangulation. **Results**: Participants reported three dimensions of health awareness. (1) App-enabled accessibility as a catalyst for physical health awareness (i.e., physical health benefits) included improved vitality and increased motivation to maintain exercise routines. (2) App-based planning and anticipation in supporting mental well-being (i.e., mental health benefits) involved stress reduction, emotional recovery, enjoyment, and heightened self-care awareness. (3) Platform-mediated social encounters and the construction of social health (i.e., social health benefits) reflected expanded social networks, strengthened interpersonal relationships, and a greater sense of belonging fostered through shared leisure experiences. **Conclusions**: Smartphone-based leisure platforms play a meaningful role in enhancing users’ awareness of multidimensional health benefits. By improving accessibility, diversifying leisure options, and facilitating social interaction, these platforms support holistic well-being. The findings contribute to understanding technology-mediated leisure and offer practical implications for designing digital leisure services that promote physical, mental, and social health.

## 1. Introduction

Technology has continuously contributed to the growing use of leisure-related platforms, reshaping how people engage in leisure activities. In South Korea, leisure activities involving smart devices have markedly increased, especially during the pandemic. Recent national data from South Korea indicate that 55.3% of total leisure time was spent watching sports and 69.1% was spent using leisure facilities [1], reflecting the steady integration of smartphones into everyday leisure. Rather than directly producing health outcomes, smartphone-based platforms increasingly facilitate access to leisure activities that are associated with physical, mental, and social health benefits [2,3,4]. These trends suggest that digital platforms are becoming an integral part of everyday leisure participation. As concerns about physical inactivity, mental health challenges, and social isolation continue to grow, understanding how leisure activities contribute to health and well-being has become increasingly important [5]. In this context, leisure participation is no longer viewed merely as discretionary time use, but as a meaningful resource that can support holistic health across physical, mental, and social dimensions.

Engagement in leisure activities has been widely associated with physical, mental, and social health benefits, including improved physical functioning, psychological well-being, and social connectedness [6,7]. Alongside these established benefits, smartphone-based leisure reservation applications have further transformed leisure participation by increasing accessibility, reducing participation barriers, and facilitating informed decision-making through real-time information and user-generated feedback [8,9,10]. Prior research on these platforms has primarily examined perceived usefulness and user satisfaction in relation to leisure participation [11,12].

More broadly, existing studies on technology-mediated leisure have investigated mobile fitness applications, travel platforms, and digital booking systems in relation to participation patterns, satisfaction, and behavioral intentions (e.g., [13,14]). However, much of this research has relied on quantitative approaches and has provided limited insight into how individuals subjectively experience and interpret health and well-being benefits within these technology-mediated leisure contexts. As a result, relatively little research has explored how engagement through such applications shapes individuals’ lived experiences and perceptions of health and well-being.

Accordingly, the purpose of this study is to examine how individuals experience and interpret physical, mental, and social health benefits that emerge through engagement with smartphone-based leisure reservation platforms. While existing leisure and health literature has established the positive outcomes of leisure participation [8], prior research has largely focused on participation itself or on the perceived usefulness of digital platforms, offering limited insight into how health benefits are experienced and constructed within technology-mediated leisure contexts. By adopting an interpretative phenomenological approach (IPA), this study advances existing knowledge by shifting attention from outcome-based explanations to users’ lived experiences, and by conceptualizing mobile platforms not merely as facilitators of leisure participation, but as active mediators that shape health awareness and meaning-making in contemporary leisure engagement. This study addresses the following research question:

**Research Question:** How do individuals experience and make sense of physical, mental, and social health benefits through engagement with smartphone-based leisure reservation platforms?

## 2. Literature Review

### 2.1. Leisure Activities and Health Benefits

Leisure activities are broadly defined as self-determined and enjoyable activities undertaken during one’s free time, serving psychological, physical, and social functions [15,16]. Because leisure activities are essential for fulfilling basic psychological needs and maintaining overall life satisfaction, engaging in them can have substantial effects on both physical and psychological health [17]. Empirical research has consistently shown that participation in leisure activities contributes to stress reduction, improved mood, and enhanced overall well-being [18,19]. These benefits manifest across physical, psychological, and social domains, as leisure engagement not only enhances physical fitness but also fosters emotional stability and social connectedness [6]. In recent years, the growing integration of digital technologies has further transformed how individuals engage in leisure, offering new ways to experience and sustain its health-related benefits. While these studies have firmly established the health-promoting potential of leisure participation, much of this literature has conceptualized health benefits as relatively stable outcomes of engagement. Consequently, less attention has been given to how these benefits are subjectively experienced, interpreted, and made meaningful by individuals—particularly within digitally mediated leisure contexts.

### 2.2. Technology Integration in Modern Leisure Activities

Technological advancement has profoundly enhanced the quality of human life [20]. Innovations in communication, mobility, and information access have made everyday tasks more efficient and enriched the way people connect, learn, and experience the world. In particular, digital technologies have allowed individuals to overcome temporal and spatial barriers, expanding opportunities for education, work, and recreation [8,9]. As technology has become deeply embedded in daily routines, its influence has also extended to leisure—a domain traditionally associated with rest and self-fulfillment. The integration of technology into leisure contexts has enabled individuals to access and personalize various activities more conveniently than ever before, offering new ways to pursue relaxation, enjoyment, and social engagement [12,13,20].

Technology has dramatically transformed how individuals participate in leisure activities. Mobile applications and online platforms have enhanced the accessibility and convenience of leisure by allowing users to discover, plan, and engage in activities anytime and anywhere [12]. This digital flexibility has increased both the frequency and spontaneity of participation, as people can now pursue recreational experiences without spatial or temporal limitation. Moreover, technological systems such as data-driven recommendation algorithms and gamified feedback mechanisms have personalized leisure participation, tailoring activities to users’ unique interests and motivational states [9,21]. Beyond individual engagement, technology has also expanded social connectivity through digital communities and social media, enabling people to share experiences and maintain relationships that transcend physical boundaries [22]. However, existing research has largely emphasized these functional advantages of technology—such as accessibility, personalization, and connectivity—while paying comparatively less attention to how such technological features shape individuals’ subjective experiences of leisure and health. While these advancements have enriched the leisure experience, scholars have cautioned that excessive dependence on digital environments may lead to reduced spontaneity and lower intrinsic motivation [23]. Taken together, technological integration has not only redefined how leisure is accessed and experienced but also reshaped its psychological and social meanings, offering new perspectives on the relationship between technology use and health benefits.

Although prior studies have provided valuable insights into how technology enhances the accessibility and convenience of leisure participation, much of this research has also focused on usage patterns, motivations, or behavioral outcomes. Less attention has been paid to how individuals actually experience and interpret the health and well-being benefits that emerge from technology-mediated leisure engagement. In particular, the predominance of quantitative approaches has limited understanding of the subjective, contextual, and meaning-making processes through which digital leisure contributes to health and well-being. Moreover, existing studies have primarily employed quantitative approaches, offering a limited understanding of the nuanced and subjective meanings people attach to these experiences. Therefore, further exploration of individuals lived experiences is needed to capture how health applications shape the qualitative dimensions of leisure and well-being in the digital age.

## 3. Method

This study was grounded in the philosophical assumptions of constructivist/interpretivist paradigm, which aligns with the principles of IPA. This paradigm assumes a relativist ontology, recognizing that individuals experience and interpret their worlds in multiple and subjective ways, and a transactional/subjectivist epistemology, which views knowledge as co-constructed through interactions between researchers and participants [24]. Within this philosophical orientation, the study applied IPA to obtain an in-depth understanding of the experiences of individuals who regularly use smartphones to book and enjoy leisure activities. Drawing on Smith and Fieldsend’s [25] perspective, the researchers sought to explore how participants make sense of their leisure practices and the meanings they attach to their experiences. The analytical process followed the principle of double hermeneutics, in which the researcher attempts to interpret how participants themselves interpret their lived experiences. Throughout the analysis, the researcher engaged in continuous reflexivity to minimize the influence of personal preconceptions and assumptions, while prioritizing participants’ perspectives and maintaining open and reflective attitudes. This approach enabled a rich, nuanced, and contextually grounded understanding of the participants lived experiences as conveyed through their narratives [26].

### 3.1. Participants and Ethical Consideration

Consistent with the methodological orientation of IPA, this study recruited individuals who possessed direct and meaningful experience with smartphone-based leisure activities reservation. A combination of purposive and snowball sampling was used to identify participants capable of providing rich and reflective accounts relevant to the research purpose. Participants recruitment followed three criteria. First, adults of any gender, education level, occupation, income, or religious background were eligible. Second, participants were required to have regularly used a smartphone leisure-activity platform to reserve their activities for at least one year, to have made a minimum of three actual bookings, and to maintain active personal social media accounts. Third, to ensure diversity in experiential perspectives, individuals whose leisure activities were concentrated in only one type of activity were excluded.

All procedures adhered to established ethical research guidelines and were approved by the Institutional Review Board of the authors’ affiliated institution. Prior to data collection, participants received a comprehensive explanation of the study’s aims, interview procedures, potential risks and benefits, and confidentiality safeguards. Written informed consent was obtained from each participant, confirming their voluntary participation and understanding of their rights. All signed consent forms were securely stored in accordance with ethical standards.

### 3.2. Data Collection and Analysis

This study employed in-depth, semi-structured interviews as the primary method of data collection, focusing on participants’ health perceptions, perceived benefits, and shifts in awareness associated with booking and engaging in leisure activities through smartphone applications. All interviews were conducted at locations chosen by the participants and consisted of two sessions per individual, each lasting approximately 60 min. To foster rapport and facilitate open communication, the researcher maintained periodic contact with participants through social media between interview sessions. Following the first round of interviews, all audio-recorded data were transcribed verbatim and used to inform the development of follow-up questions for the second round. Data collection occurred over a three-month period, from June to September 2025. The verbatim quotations presented in the Section 4 are drawn from these interview transcripts and are identified using pseudonyms to protect participants’ anonymity.

Data were analyzed using IPA, following the methodological guidelines outlined by [23]. The analysis involved iterative engagement with the transcripts, including multiple readings, initial noting, the development of emergent themes, and the identification of patterns across cases. Throughout the analytical process, the researcher sought to remain reflexive, prioritizing participants’ subjective meanings and interpretations while acknowledging the interpretive role inherent in IPA’s double-hermeneutic approach. The researcher’s academic background in sport management and leisure studies informed familiarity with the research context; however, reflexive practices, including analytic memo writing and peer debriefing, were employed to critically examine assumptions and minimize undue influence on data interpretation.

The sample size was considered appropriate for IPA, which emphasizes in-depth, idiographic analysis of a relatively small and homogeneous sample. Data collection continued until thematic saturation was reached, as no substantively new themes emerged in the later stages of analysis, allowing for rich and detailed exploration of participants’ lived experiences [26].

To enhance the trustworthiness of the study, several strategies were employed throughout the research process. Member checking was conducted to improve the credibility of the findings [25]. Transcripts of the in-depth interviews were shared with participants via mobile message application (e.g., KakaoTalk), which is the most widely used instant messaging platform in South Korea, allowing them to review the accuracy of the content and provide feedback on the researcher’s interpretations.

During the subsequent stages of analysis, peer debriefing was utilized to examine the appropriateness of the emergent themes and analytic decisions. Colleagues familiar with qualitative research reviewed portions of the data and provided critical feedback, contributing to the dependability of the findings [24]. In addition, triangulation was applied across different stages of data collection and analysis to strengthen confirmability. This included cross-checking interview data, analytic memos, and field notes to ensure consistency and reduce the influence of researcher bias [24]. Through these combined procedures, the study sought to establish a rigorous and trustworthy qualitative research process.

## 4. Results

This study examined how individuals who regularly use smartphone-based leisure reservation platforms perceive the health benefits associated with their leisure activities. Demographic information and the primary type of leisure platform used by each participant are presented in Table 1. The analysis revealed that participants attributed a wide range of health-related outcomes to their engagement in these activities. Participants’ perceptions of health benefits were reflected in three interconnected dimensions: (1) app-enabled accessibility as a catalyst for physical health awareness, (2) app-based planning and anticipation in supporting mental well-being, and (3) platform-mediated social encounters and the construction of social health.

### 4.1. App-Enabled Accessibility as a Catalyst for Physical Health Awareness

Participants reported that frequent use of leisure reservation platforms heightened their awareness of physical health, emphasizing that this awareness stemmed less from the physical activity itself than from the platform’s accessibility features—such as the ease of locating available facilities and booking activities without temporal or spatial constraints. Many participants indicated that the convenience of booking exercise spaces or travel-related activities via smartphone platforms served as a key motivator for continued engagement. Across accounts, platform-enabled accessibility emerged as a central mechanism facilitating sustained physical activity and increased physical vitality. Several participants described experiencing positive physical changes after engaging in leisure activities arranged through these platforms. Bella, for example, shared how the platform enabled her to resume exercise routines disrupted during the pandemic:

“Before the pandemic era, I was able to maintain a healthy life through workouts at the gym. I used to take time out every day to exercise, but since the pandemic, I have not been able to exercise much. During this time, I found a place to exercise through the leisure reservation platform, and after exercising, I felt physically healthy.”

This account highlights how the platform’s function of enabling quick identification and reservation of exercise spaces reduced participation barriers, thereby facilitating more consistent engagement with physical activity. Participants consistently described leisure reservation platforms as enabling them to meet their physical health needs more effectively by simplifying access to diverse activity options, including recreational sports, guided lessons, and travel-related physical activities. James, a water-sports enthusiast, explained:

“When life gets tough and challenging, I often turn to water sports to help me stay healthy. I enjoy paddleboarding on the river near me, or if I can afford it, I go to places like Yangyang County or Jeju Island to take surfing lessons. Using a leisure sports platform makes it easier for me to enjoy these activities. I often feel physically fitter after doing them.”

Thus, perceived physical health benefits were not attributed solely to the activities themselves, but to the platform’s role in simplifying access and reducing logistical constraints. Overall, participants perceived leisure activities booked through these platforms as contributing significantly to their physical well-being, particularly by enabling the integration of physical activity into otherwise constrained schedules, such as weekends or vacation periods.

### 4.2. App-Based Planning and Anticipation in Supporting Mental Well-Being

Participants who regularly used leisure reservation platforms emphasized the mental health benefits associated with engaging in leisure activities, particularly in relation to stress management and psychological recovery. Leisure participation was widely described as an effective strategy for alleviating work-related fatigue and emotional strain. Alexander, for example, described how booking weekend water-skiing lessons through a leisure platform supported his mental well-being:

“Recently, I have been working a lot of business trips and overtime because of my increased workload, which caused me lots of fatigue and made my life very difficult. I wanted to get some exercise, so I decided to learn water skiing, which I can do every weekend. I signed up for lessons through a leisure booking platform and learned water skiing, and I felt my mental health was restored.”

Participants further noted that these mental health benefits were closely linked to the platform’s reservation and planning functions, which enabled them to secure leisure time in advance and reduced uncertainty surrounding participation. By structuring leisure engagement ahead of time, platforms helped participants maintain consistent access to restorative activities. Bella, an office worker, described how booking cultural leisure activities through a platform helped her decompress after demanding workdays:

“I felt myself de-stressing during the few hours I spent visiting art galleries and viewing famous works during my leisure time.”

Across participants, leisure engagement was described as fostering emotional balance, reduced anxiety, and psychological recovery from daily stressors. Some also highlighted relational dimensions of mental well-being, noting that platform-facilitated leisure activities supported emotional connection and relational repair within family contexts.

A notable theme was increased self-care awareness among platform users. Participants reported that engaging in leisure activities prompted greater recognition of the importance of rest, recovery, and personal maintenance. James explained:

“Working out at the gym is a way to improve my mental health. Working out without missing a day is closely related to my mental health. I often use leisure booking platforms to exercise, which has helped me to overcome various constraints in leisure activities.”

This reflects how platform-based planning and scheduling affordances alleviated practical constraints, supporting mental well-being by enabling sustained self-care routines. Across these accounts, participants emphasized that the platform’s planning and scheduling functions were essential in making restorative leisure experiences feasible within their everyday routines. Overall, improvements in mental well-being—including stress reduction, emotional balance, enjoyment, and self-care awareness—emerged not only from leisure participation itself, but from the platform’s ability to structure anticipation, continuity, and engagement.

### 4.3. Platform-Mediated Social Encounters and the Construction of Social Health

Participants described experiencing notable social health benefits through leisure activities booked via leisure reservation platforms. These benefits were consistently attributed to platform-mediated features, such as group-based bookings and shared participation formats, which facilitated interaction among individuals with common leisure interests. Participants emphasized that such features created opportunities to meet new people, interact with unfamiliar individuals, and develop social connections beyond routine daily interactions. Gabriel articulated this experience clearly:

“I was very surprised to realize that after experiencing a tour with others on a leisure booking platform, I felt a special social connection with people I had never met before.”

This illustrates how platform-mediated leisure participation created opportunities for social connection that may not have emerged through individually arranged leisure activities. Participants further noted that shared leisure interests enabled the formation of social bonds even during short-term interactions.

Several participants highlighted the role of leisure platforms in expanding social networks. Platform-facilitated group tours, travel communities, and activity-based gatherings enabled participants to engage with new peer groups and broaden their social circles. Iris described the transition from anonymous online interaction to meaningful face-to-face engagement:

“Sharing experiences or providing information on leisure booking platforms is done anonymously. However, when you actually use the platform, you can meet new people and engage in leisure activities. When you also exercise or travel with people having similar interests, you will experience different kinds of pleasure without even realizing it.”

Such accounts demonstrate how digital platforms functioned as social intermediaries, transforming anonymous online interactions into meaningful in-person experiences. Participants further emphasized that platform-mediated group arrangements fostered cooperative interactions, facilitating trust-building and mutual support even among individuals meeting for the first time. These interactions contributed to enhanced social well-being, including a sense of belonging, increased self-esteem, and heightened awareness of social health.

Overall, leisure activities facilitated through reservation platforms were perceived as powerful social experiences that enabled interaction, fostered belonging, and strengthened interpersonal relationships. These social health benefits were thus constructed through the platform’s mediation of interaction, rather than arising solely from leisure participation itself.

## 5. Discussion

This study examined how the use of smartphone-based leisure reservation platforms enhances individuals’ awareness of health benefits across three dimensions: physical, mental, and social health. Guided by the research question of how individuals experience and make sense of health benefits through engagement with smartphone-based leisure reservation platforms, this Discussion focuses on interpreting how platform-specific features shape health awareness beyond leisure participation itself. The findings offer several important insights. First, the findings indicate that leisure engagement facilitated through smartphone-based reservation platforms plays a meaningful role in shaping mental health awareness. Rather than leisure participation alone, the platform-supported structuring of leisure—through planning, anticipation, and reduced uncertainty—appears to contribute to stress reduction, emotional recovery, and psychological balance. This interpretation aligns with prior research demonstrating that leisure engagement enhances resilience and mental well-being [27,28], while extending this literature by highlighting how digital platforms actively mediate these outcomes. In particular, the ability to plan and secure leisure activities in advance provided users with a sense of purpose, structure, and control, which contributed to greater emotional stability and overall life satisfaction.

Second, the findings suggest that smartphone-based leisure reservation platforms contribute to heightened physical health awareness by lowering participation barriers and supporting sustained engagement in physical activities. Consistent with previous research on technology-assisted leisure and health behavior change [29], participants’ experiences indicate that platform-enabled access to diverse activity options encouraged regular participation and reinforced recognition of physical health benefits, including improved cardiovascular functioning and enhanced physical fitness [30,31,32]. Importantly, the flexibility afforded by these platforms allowed individuals to select activities aligned with their abilities and schedules, thereby sustaining motivation and supporting long-term physical health management [6,33].

Third, the findings demonstrate that leisure activities booked through reservation platforms significantly shape social health awareness by facilitating social interaction and integration. Platform-mediated group activities and shared participation formats functioned as social catalysts, enabling users to form new social connections and transition from online coordination to meaningful offline engagement. These processes reflect established understandings of leisure as a context for community participation and social bonding [7], while further illustrating how digital platforms actively structure opportunities for belonging, relational confidence, and social well-being. In this sense, social health benefits emerged not merely from leisure participation itself, but from the platform’s role in mediating and sustaining social encounters.

### 5.1. Theoretical Contributions and Practical Implications

This research provides valuable insights for academics as well as practitioners. First, from a theoretical viewpoint, this study advances leisure and health scholarship by demonstrating that smartphone-based leisure reservation platforms actively mediate users’ motivation and health awareness, rather than merely facilitating access to leisure activities. In contrast to traditional booking methods, which often require advance planning, limited information, or offline coordination, the digital interface of smartphone-based leisure reservation platforms appeared to alter users’ motivation toward leisure participation [14]. Participants described being more inclined to engage in leisure activities when options were immediately visible, easily comparable, and reservable with minimal effort. This immediacy reduced psychological barriers to participation and transformed leisure engagement from a deliberate, effortful decision into a more spontaneous and achievable action. As a result, the platform did not simply function as a booking tool but played an active role in shaping users’ motivation by lowering participation thresholds and sustaining intention through planning and anticipation. Building on this, the present study expands the conceptualization of health benefits associated with leisure participation by illustrating how platform-mediated motivation translates into enhanced awareness of physical, mental, and social health benefits. While prior research has largely emphasized single dimensions of health outcomes (e.g., psychological or emotional benefits) of using leisure reservation platforms [34,35], or focused on users’ future behavioral intentions [8], our findings offer a multidimensional framework that integrates digital leisure behavior into contemporary understandings of health and well-being.

Second, the study advances qualitative leisure scholarship by illustrating the value of IPA in examining technology-mediated leisure experiences. While previous studies have primarily relied on quantitative approaches to examine the effects of mobile applications on leisure participation and behavioral intentions (e.g., [13,36]), such work has provided limited insight into how digital platforms actively shape users’ meaning-making processes. By applying IPA, the present findings reveal that smartphone-based leisure reservation platforms function not merely as facilitators of participation, but as mediating mechanisms that amplify health awareness through accessibility, planning and anticipation, and platform-mediated social interaction. In doing so, this study extends prior leisure and health research by shifting attention from outcome-based explanations to the experiential and interpretive processes through which health benefits are understood.

The findings of this study offer concrete implications for the design and deployment of smartphone-based leisure reservation platforms and related marketing strategies. First, platform designers can promote holistic well-being by intentionally aligning specific digital features with users’ health-related experiences. For example, enhanced accessibility features, such as real-time availability and simplified booking interfaces, can lower participation barriers and encourage spontaneous physical activity among users with limited time. Planning and scheduling functions, including advance reservations and reminders, may support stress management by enabling users to anticipate leisure time and integrate restorative activities into their routines. In addition, community-based and group-oriented features, such as shared bookings or interest-based activity groups, can facilitate social interaction and foster a sense of belonging, thereby strengthening users’ social health awareness.

Second, smartphone-based leisure reservation platforms have the potential to play a meaningful role in enhancing public health and broader community wellness initiatives. Given this consideration, platform providers could collaborate with local governments or community organizations to promote affordable and accessible leisure programs tailored to diverse populations, such as office workers, families, or older adults. For instance, partnerships could focus on highlighting low-cost physical activities, nature-based programs, or group leisure opportunities that reduce social isolation. By strategically leveraging platform features that enhance accessibility, planning, and social connection, these collaborations can support sustained engagement in health-promoting leisure behaviors and contribute to broader public health goals.

### 5.2. Limitations and Future Research

Although this study offers meaningful insights, it also has several limitations. First, this study employed a cross-sectional qualitative design, which limits the ability to examine how perceptions of health benefits may evolve over time with continued platform use. As a result, it is not possible to determine whether the identified health awareness emerged as a consequence of platform use or whether participants with pre-existing health orientations were more likely to engage with such platforms.

Second, the findings are situated within the South Korean context, where digital infrastructure and mobile platform usage are highly developed. Cultural norms surrounding leisure participation, technology adoption, and social interaction may have shaped participants’ experiences, suggesting that similar platforms could be experienced differently in cultural or institutional contexts with lower levels of digital integration.

Third, the participant sample was relatively homogeneous in terms of socioeconomic characteristics, with most participants identified as employees or self-employed individuals. This homogeneity may be partly attributable to the use of snowball sampling, which can lead to the recruitment of participants with similar social and economic backgrounds. Given that regular use of smartphone-based leisure reservation platforms often requires discretionary income and engagement in paid leisure activities, the experiences captured in this study may disproportionately reflect the perspectives of middle- to higher-income individuals.

Finally, while the study does not aim for statistical generalization, the context-specific nature of the sample may limit the transferability of findings to other populations or settings. Readers are therefore encouraged to consider similarities and differences in platform design, cultural norms, and leisure practices when applying these insights to other contexts.

## 6. Conclusions

This study aims to explore how smartphone-based leisure reservation platforms influence individuals’ awareness of physical, mental, and social health benefits. The findings showed that platform-enabled leisure activities enhanced users’ understanding of holistic well-being by improving access to physical activities, supporting stress relief and emotional recovery, and facilitating meaningful social interactions. These results highlight the growing role of digital platforms in shaping leisure experiences and promoting multidimensional health. As smartphone-based leisure services continue to expand, this research provides comprehensive insights suggesting that appropriate use may serve as a valuable resource for supporting healthier and more fulfilling lifestyles.

## Figures and Tables

**Table 1:** Demographic Characteristics of the Participants.

	Pseudonym	Sex	Age	Occupation	Primary Type of Leisure Platform Used	Leisure Reservation Platforms Usage Count (Month)
1	Alexander	M	35	Employee	Sport- and activity-focused leisure platform	4
2	Bella	F	35	Employee	General leisure reservation platform	3
3	Charles	M	27	Self-employed	Travel- and experience-oriented leisure platform	5
4	Daniel	M	36	Self-employed	Sport-specific leisure platform	3
5	Emily	F	33	Self-employed	General leisure reservation platform	5
6	Fiona	F	32	Employee	Travel- and experience-oriented leisure platform	3
7	Gabriel	M	30	Employee	Travel- and experience-oriented leisure platform	5
8	Hannah	F	29	Employee	General leisure reservation platform	4
9	Iris	F	31	Self-employed	General leisure reservation platform	5
10	James	M	34	Self-employed	Sport- and activity-focused leisure platform	4

## Data Availability

The data presented in this study are available on request from the corresponding author. The data are not publicly available based on the IRB restrictions.

## Abbreviation

The following abbreviation is used in this manuscript:

IPA	Interpretative Phenomenological Analysis
